# Efficacy and safety of three new oral antiviral treatment (molnupiravir, fluvoxamine and Paxlovid) for COVID-19：a meta-analysis 

**DOI:** 10.1080/07853890.2022.2034936

**Published:** 2022-02-04

**Authors:** Wen Wen, Chen Chen, Jiake Tang, Chunyi Wang, Mengyun Zhou, Yongran Cheng, Xiang Zhou, Qi Wu, Xingwei Zhang, Zhanhui Feng, Mingwei Wang, Qin Mao

**Affiliations:** a Hangzhou Institute of Cardiovascular Diseases, Affiliated Hospital of Hangzhou Normal University, Hangzhou, PR China; b Hangzhou Normal University, Hangzhou, PR China; c Department of Molecular and Cellular Physiology, Shinshu University School of Medicine, Matsumoto, Japan; d School of Public Health, Hangzhou Medical College, Hangzhou, PR China; e Department of Neurology, Affiliated Hospital of Guizhou Medical University, Guiyang, PR China

**Keywords:** COVID-19, molnupiravir, fluvoxamine, Paxlovid^™^

## Abstract

**Background:**

The coronavirus disease (COVID-19) epidemic has not been completely controlled. Although great achievements have been made in COVID-19 research and many antiviral drugs have shown good therapeutic effects against COVID-19, a simple oral antiviral drug for COVID-19 has not yet been developed. We conducted a meta-analysis to investigate the improvement in mortality or hospitalization rates and adverse events among COVID-19 patients with three new oral antivirals (including molnupiravir, fluvoxamine and Paxlovid).

**Methods:**

We searched scientific and medical databases, such as PubMed, Web of Science, Embase and Cochrane Library for relevant articles and screened the references of retrieved studies on COVID-19.

**Results:**

A total of eight studies were included in this study. The drug group included 2440 COVID-19 patients, including 54 patients who died or were hospitalized. The control group included a total of 2348 COVID-19 patients, including 118 patients who died or were hospitalized. The overall odds ratio (OR) of mortality or hospitalization was 0.33 (95% confidence interval [CI], 0.22–0.49) for COVID-19 patients in the drug group and placebo group, indicating that oral antiviral drugs were effective for COVID-19 patients and reduced the mortality or hospitalization by approximately 67%.

**Conclusions:**

This study showed that three novel oral antivirals (molnupiravir, fluvoxamine and Paxlovid) are effective in reducing the mortality and hospitalization rates in patients with COVID-19. In addition, the three oral drugs did not increase the occurrence of adverse events, thus exhibiting good overall safety. These three oral antiviral drugs are still being studied, and the available data suggest that they will bring new hope for COVID-19 recovery and have the potential to be a breakthrough and very promising treatment for COVID-19.

**KEY MESSAGES**
- Many antiviral drugs have shown good therapeutic effects, and there is no simple oral antiviral drug for COVID-19 patients.
- Meta-analysis was conducted for three new oral antivirals to evaluate the improvement in mortality or hospitalization rates and adverse events among COVID-19 patients.
- We focussed on three new oral Coronavirus agents (molnupiravir, fluvoxamine and Paxlovid) and hope to provide guidance for the roll-out of oral antivirals.

## Introduction

In December 2019, COVID-19 caused by severe acute respiratory syndrome coronavirus type 2 (SARS-COV-2) broke out in China. The COVID-19 epidemic has rapidly spread across the globe, with 251,788,329 cases of COVID-19 and 5,077,907 deaths reported as of 12 November 2021 [1]. Development of a vaccine against the COVID-19 virus has continued, and mass vaccination campaigns are still going on. A study in Italy has shown that COVID-19 vaccine can effectively reduce the mortality of COVID-19 infected patients [2]. As of 12 November 2021, more than 2.3 billion novel coronavirus vaccine doses were given to people in China [3]. However, studies have shown that even vaccinated people are infected with novel coronavirus variants. Millions of immunocompromised patients may not be fully protected after vaccination, and existing vaccines may not be effective against new novel coronavirus variants [4,5]. Although an antiviral drug Remdesivir has been developed that has shown good effects in antiviral therapy [6], some clinical trials have not fully demonstrated its beneficial effects on SARS-COV-2; moreover, the drug is expensive and must be administered intravenously in a hospital setting [6–8]. Therefore, it is essential to develop simple oral coronavirus drugs.

Recently, three new oral coronavirus drugs have shown effective results in clinical studies. Recently, molnupiravir, an orally active RdRp inhibitor with a favourable pharmacokinetic profile, has received considerable attention owing to its ability to inhibit SARS-COV-2 replication, remove SARs-COV-2 rapidly, reduce viral load and recover fast [4]. Molnupiravir is the isopropyl ester prodrug of the ribonucleoside analogue β-D-N4-hydroxycytidine (NHC) [9]. An *in vitro* evidence shows that molnupiravir is a potent inhibitor of SARS-CoV-2 replication with an EC50 in the submicromolar range [9–11]; the effect of this antiviral injection was also observed in animal models [9,12,13]. A study showed that the time taken for viral RNA clearance decreased, and a greater proportion of overall clearance was achieved in participants administered with molnupiravir *vs.* placebo [14]. In addition, molnupiravir has shown promising efficacy and safety in phase I/II/III clinical trials. Studies have shown that molnupiravir reduces the risk of hospitalization or death by approximately 50% in non-hospitalized adults with mild-to-moderate COVID-19 disease who are at risk for poor prognosis, and the incidence of any adverse events was comparable between the two groups (35 and 40%, respectively), as was the incidence of drug-related adverse events (12 and 11%, respectively) [15]. Fluvoxamine, another oral medication and a selective serotonin reuptake inhibitor and *σ*-1 receptor agonist [16], has shown potential of early outpatient treatment of COVID-19 in previous studies [17–19] and also good safety and effectiveness in patients in intensive care unit (ICU) [16]. Seftel and Boulware showed that no hospitalization occurred in the fluvoxamine group, while six patients out of the 48 control patients required hospital admission [17]. In addition, prospective cohort trials of fluvoxamine in ICU patients showed an overall mortality rate of 58.8% (*n* = 30/51) in the fluvoxamine group compared with 76.5% (*n* = 39/51) in the control group [16]. Paxlovid is an investigational SARS-CoV-2 protease inhibitor antiviral therapy developed by Pfizer Inc., specifically designed to be administered orally [20]. Their recent study showed that Paxlovid reduced the risk of hospitalization or death by 89% [20].

At present, clinical studies on these three oral coronavirus drugs are continuing; better results are expected. We conducted this meta-analysis to further evaluate the improvement in mortality or hospitalization rates and adverse events among COVID-19 patients with these three oral antiviral drugs (molnupiravir, fluvoxamine and Paxlovid) and hope to provide guidance for the rollout of these oral antivirals.

## Materials and methods

### Search strategy

We searched scientific and medical databases PubMed, Web of Science, Embase and Cochrane Library for relevant studies. We screened the references of the retrieved studies and restricted the language of the search to English. Following keywords were used in the search: COVID-19 (SARS-CoV-2, novel coronavirus 2019 and 2019-nCoV), molnupiravir (EIDD-2801/MK-4482), fluvoxamine and Paxlovid (PF-07321332; ritonavir).

### Inclusion and exclusion criteria

The inclusion criteria were as follows: (1) the article reported the clinical results of three oral COVID-19 drugs, including the total number of participants and the specific number of deaths or hospitalizations; (2) English literature.

The exclusion criteria were as follows: (1) irrelevant to the research direction, (2) no relevant data and (3) repeated literature.

### Data extraction

A total of eight studies were included in this study. The following data were collected: name of the first author, year of publication, name of the study drug, total number of subjects, and number or proportion of deaths or hospitalizations occurring or incidence of any other adverse events.

### Statistical analysis

All statistical analyses were performed using Review Manager version 5.2 software , and a binary controlled study was used to calculate the number of deaths or hospitalizations of COVID-19 patients in the oral antiviral group and placebo group, as well as the incidence of adverse events. Odds ratio (OR) and 95% confidence interval (CI) were used to measure the effect. The results of all the studies (OR) were aggregated using a fixed-effects model.

## Ethics statement

This study is a meta-analysis study. The ethics committee of Affiliated Hospital of Hangzhou Normal University approved all the procedures performed.

## Results

### Outcome of the electronic search

As of 12 November 2021, a total of 477 studies were obtained; 278 duplicate references, 93 unrelated references, 88 studies without relevant data and 10 non-English references were excluded. Finally, a total of eight studies were included.

### Characteristics of the included studies

**Figure 1.**
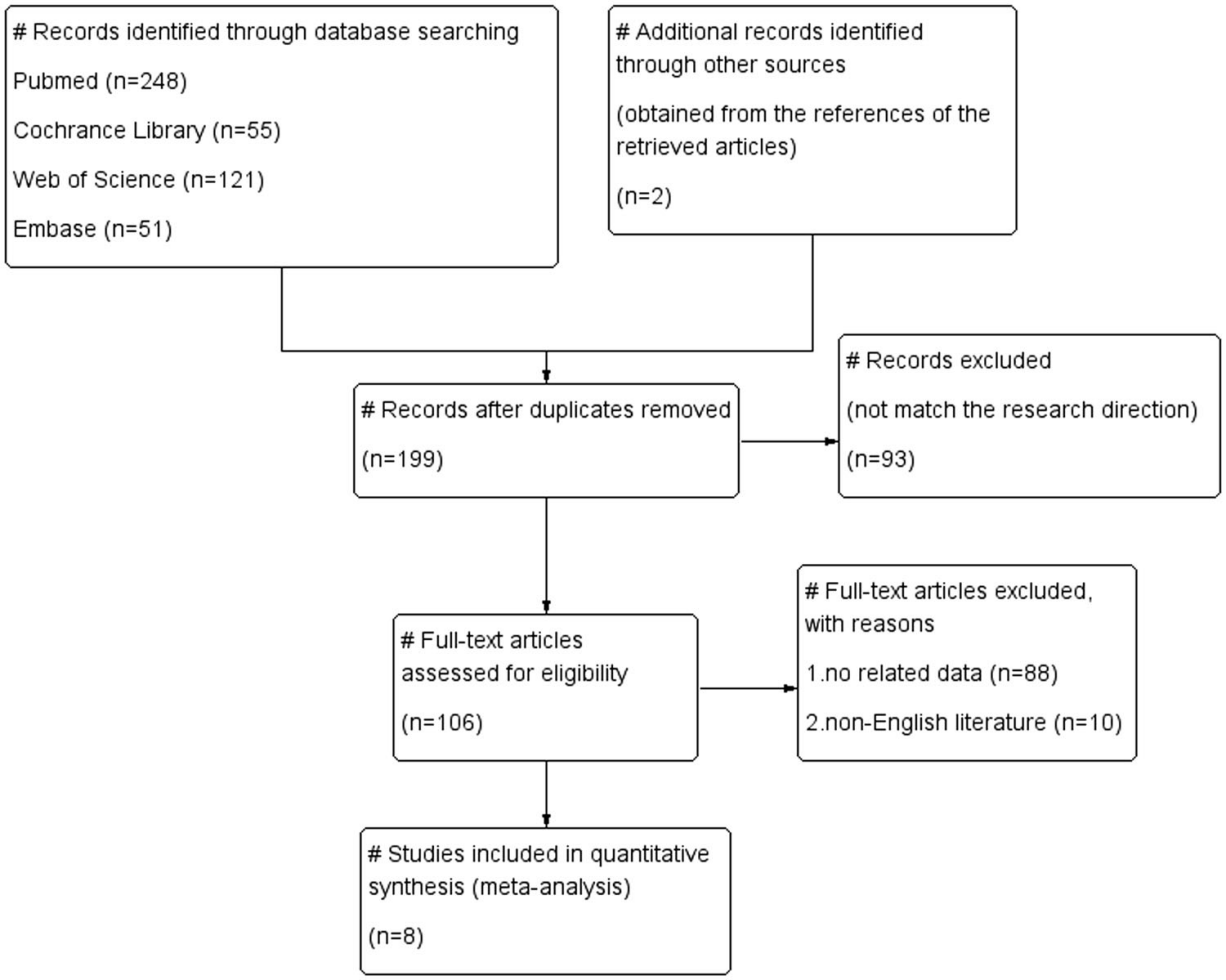
Study screening flow chart.

The characteristics of all the included literatures are shown in Table 1. Five included studies described the deaths of COVID-19 patients in the drug and control groups, and three studies described the hospitalizations of COVID-19 patients. In addition, three studies were related to molnupiravir, four studies were related to fluvoxamine and the remaining one study was related to Paxlovid. The drug group included 2440 COVID-19 patients, including 54 patients who died or were hospitalized. The control group included a total of 2348 patients, including 118 patients who died or were hospitalized (Figure 1).

**Table 1.** Basic information of the included studies.

Study	Drugs	Death or hospitalization	Drug group			Placebo group		
			Events (*n*)	Total (*n*)	Adverse events (*n*)	Events (*n*)	Total (*n*)	Adverse events (*n*)
Fischer et al. [14]	Molnupiravir	Death	0	140	42	1	62	18
Mahase [15]	Molnupiravir	Death	0	385	135	8	377	150
Calusic et al. [16]	Fluvoxamine	Death	30	51	/	39	51	/
Seftel and Boulware [17]	Fluvoxamine	Hospitalization	0	65	/	6	48	/
Lenze et al. [18]	Fluvoxamine	Hospitalization	0	80	12	6	72	18
Reis et al. [19]	Fluvoxamine	Death	17	741	169	25	756	188
Pfizer [20]	Paxlovid	Death	0	607	10	10	612	40
Hetero [21]	Molnupiravir	Hospitalization	7	371	/	23	370	/

### Meta-analysis

**Figure 2.**
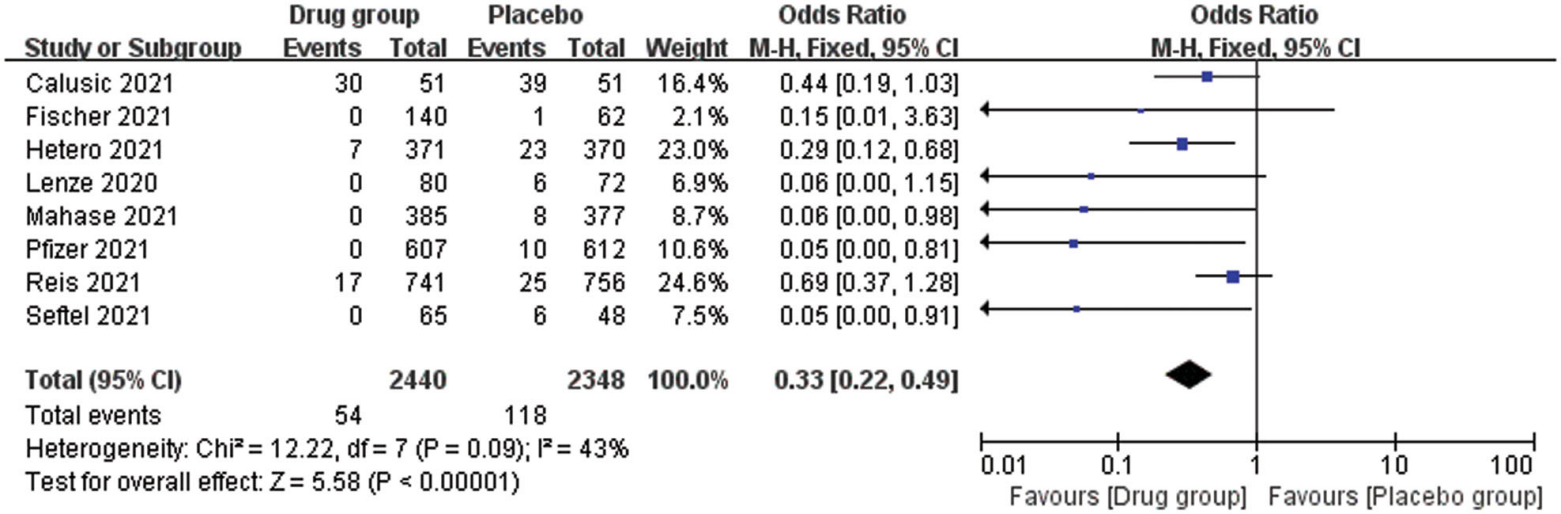
Analysis of overall death or hospitalization rates between the oral antiviral group and the placebo group.

**Figure 3.**
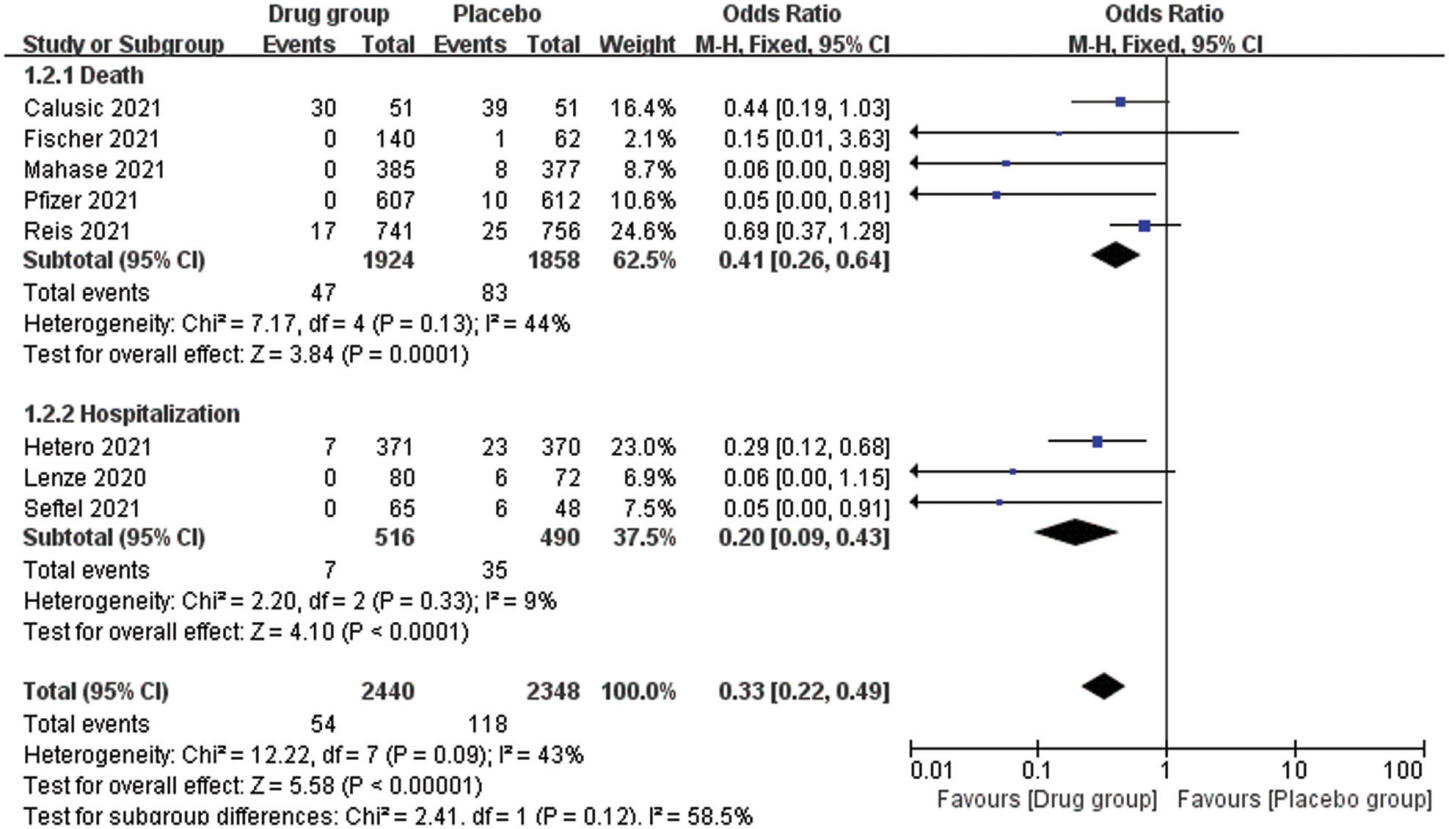
Subgroup analysis: impact of oral antiviral drugs on mortality and hospitalization rates of COVID-19 patients.

**Figure 4.**
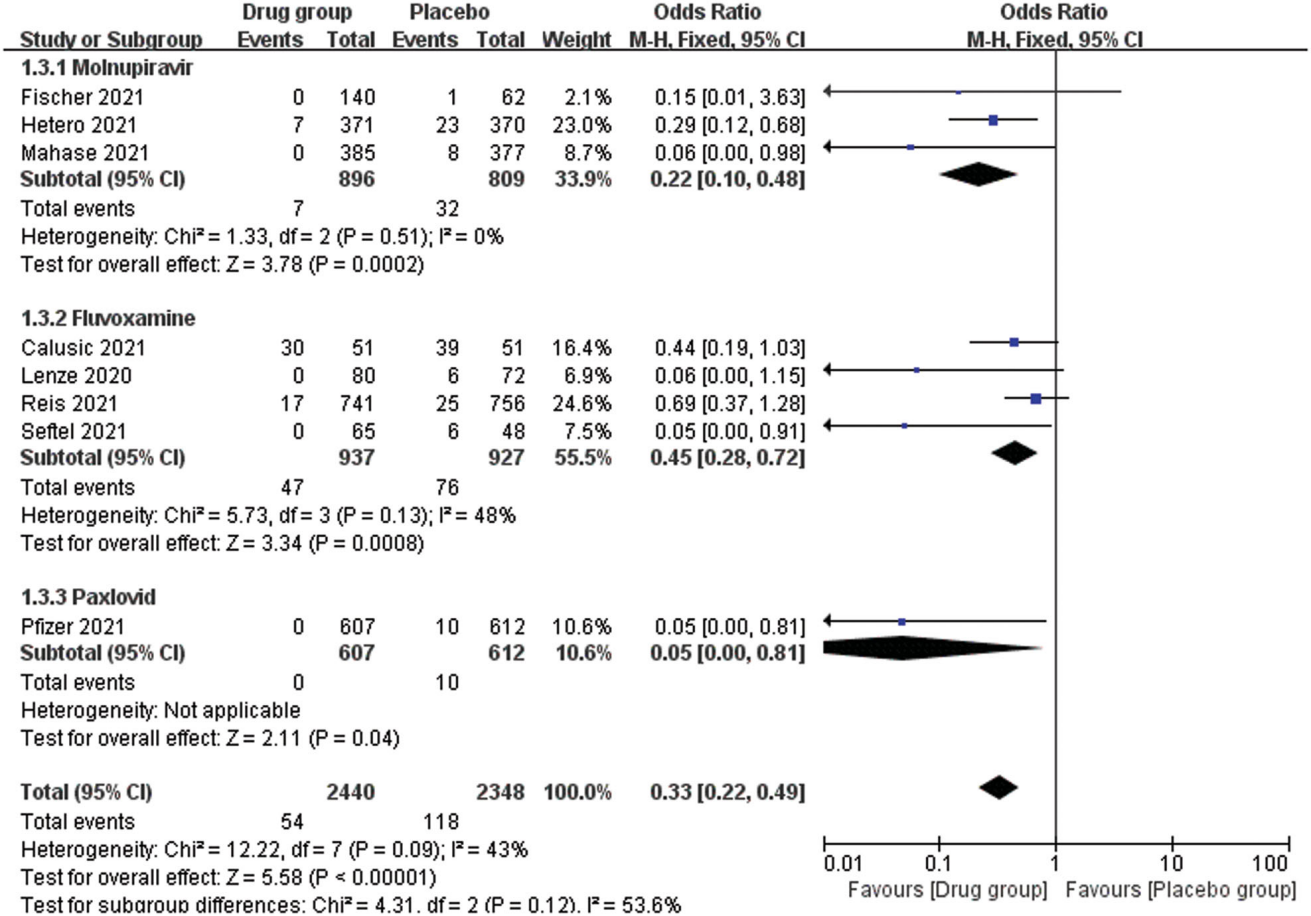
Subgroup analysis: impact of different oral antiviral drugs on mortality or hospitalization rate of COVID-19 patients.

Our study showed that the overall OR for death or hospitalization among COVID-19 patients in the drug *vs*. placebo group was 0.33 (95% CI, 0.22–0.49; *I*^2^ = 43%), *p* < .00001. This indicates that the oral antiviral drugs are effective for COVID-19 patients, reducing the mortality or hospitalization rate by approximately 67% (Figure 2). Figure 3 shows that the OR of mortality for COVID-19 patients in the drug *vs.* placebo group was 0.41 (95% CI, 0.26–0.64; *I*^2^ = 44%), *p* = .0001, indicating a 56% reduction in mortality. The OR for hospitalization was 0.20 (95% CI, 0.09–0.43; *I*^2^ = 9%), *p* < .0001, i.e. approximately 80% reduction in hospitalization rate. In addition, we analysed the efficacy of three different antiviral drugs for COVID-19 patients, and the OR was 0.22 (95% CI, 0.10–0.48) in the molnupiravir group, 0.45 (95% CI, 0.28–0.72) in the fluvoxamine group and 0.05 (95% CI, 0.00–0.81) in the Paxlovid group (Figure 4). All the three drugs used in this study showed effective therapeutic effects.

**Figure 5.**
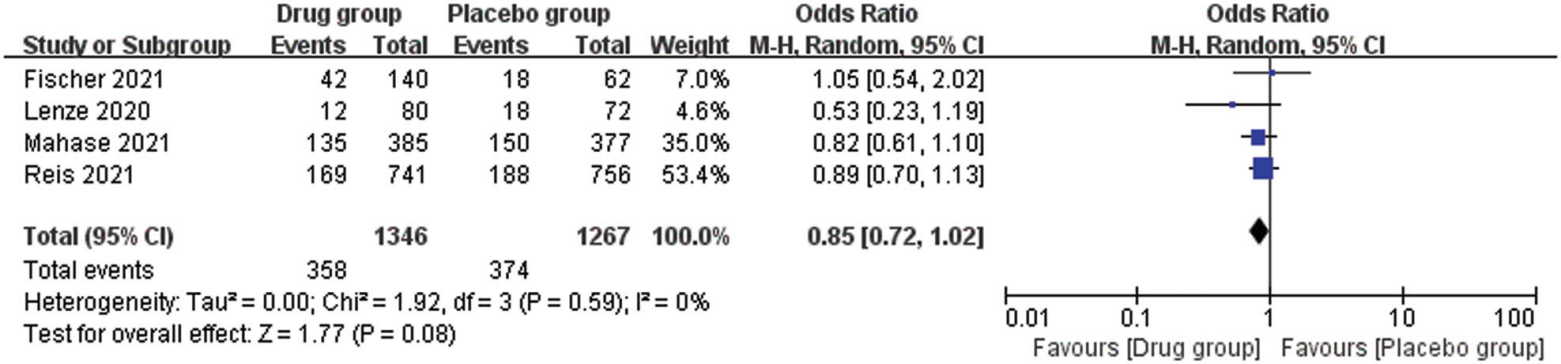
Incidence of adverse events in drug group and placebo group.

The safety of these oral antiviral drugs was analysed. The total OR of adverse events in the drug group and placebo group was 0.85 (95% CI, 0.72–1.02; *I*^2^ = 0%), *p* = .08, indicating no significant difference in the incidence of adverse events between the drug group and placebo group (Figure 5).

## Discussion

A total of eight studies were included in this study. All the three oral antiviral drugs were effective in COVID-19 patients. The overall OR of death or hospitalization for COVID-19 patients in the drug *vs.* placebo groups was 0.33 (95% CI, 0.22–0.49), indicating that these oral antiviral drugs reduced the mortality or hospitalization by approximately 67% in COVID-19 patients. The OR of mortality was 0.41 (95% CI, 0.26–0.64) for patients in the drug group and placebo group, indicating that oral medication reduced the mortality by 56%. The OR of hospitalization was 0.20 (95% CI, 0.09–0.43), indicating approximately 80% reduction in the hospitalization rate. Our study demonstrated the good therapeutic effect of these oral coronavirus drugs. The development of oral antiviral drugs is an inevitable trend in the fight against COVID-19.

At present, new oral coronavirus drugs are still being studied. Many studies have analysed how these drugs are novel coronavirus drugs. Coronaviruses are enveloped viruses. The genome of coronaviruses encodes non-structural proteins responsible for viral genome replication and transcription. Its main component is a multifunctional protein containing a central RNA-dependent RNA polymerase (RdRp) domain [9]. Studies indicate that molnupiravir, an isopropyl ester precursor, is cleaved in plasma to an active nucleoside analog NHC or eidd-1931 by host esterase [22,23]. This active form of the drug is distributed to various tissues and subsequently converted to its corresponding 5′-triphosphate (NHC triphosphate or MTP), and then the drug targets the RdRp, which is virally encoded, and competitively inhibits the cytidine and uridine triphosphates and incorporates M instead [22,24]. The RdRp uses the NHC triphosphate as a substrate instead of the cytidine and uridine triphosphates and then incorporates either A or G in the RdRp active centres, forming stable complexes and thus escaping proofreading by the synthesis of a mutated RNA [22,24]. In summary, the broad-spectrum antiviral activity of this drug can be attributed to its two-step mutagenesis mechanism [22]. In the first step, RdRp synthesizes a negative-strand genomic RNA(−gRNA) using a positive-strand genomic RNA(+gRNA) as the template [13,22,23]. In the second step, +gRNA or sub genomic RNA is synthesized using M-containing RNA as the template. The M-containing RNA in the − gRNA causes mutation in + gRNA, and subgenomic RNA is subsequently formed, resulting in mutagenesis, which is lethal to the virus [13,22,23]. Molnupiravir is only administered orally for a short period (5 d), and it is easier to administer molnupiravir on an outpatient basis and therefore has better compliance [22]. In addition, the available data indicate that molnupiravir is well tolerated and safe, at least in the short term, without any major adverse events in current clinical trials. However, one study identified mutations in molnupiravir-treated cells in animal cell cultures [25]. Although the short-term use of 5 d seems unlikely to produce mutations [22], such mutations raise concerns about whether the long-term use of molnupiravir could lead to changes in human DNA.

The potential mechanism of fluvoxamine for the treatment of COVID-19 is still uncertain; some hypotheses have been proposed. A study reported that fluvoxamine exhibited the strongest activity among all SSRIs with low nanomolar affinity on sigma-1 receptor (S1R), which may reduce the excessive inflammatory state induced by novel coronavirus by regulating S1R [26]. In addition, S1R has other antiviral effects, including reducing platelet aggregation, reducing mast cell degeneration, interfering with endolysosomal virus transport, regulating myositol requiring enzyme 1α driven inflammation and increasing melatonin levels [26], which may be important mechanisms influencing COVID-19 treatment. In addition, Paxlovid is an oral antiviral drug candidate for SARS-CoV-2 protease inhibitors, recently released by Pfizer [20]. Paxlovid is a combination of PF-07321332 and Ritonavir. Paxlovid does not work as well if it is taken on its own. The body’s defence mechanisms will remove anything that it does not recognize, including drugs, which can be digested by the liver enzymes [27]. Among them, Paxlovid is designed to block the activity of SARS-COV-2-3Cl protease, which is needed for the coronavirus to replicate [20]. Use of Paxlovid in combination with a low dose of ritonavir helps slow down the metabolism or breakdown of PF-07321332 so that it remains active in the body for longer at higher concentrations and helps in fighting the virus [20]. Paxlovid^™^ has been designed with the novel coronavirus-specific protease in mind and is therefore more specific to this coronavirus than molnupiravir [27]. Data obtained from a larger cohort of 1881 patients in EPIC-HR showed that treatment-emergent adverse events were comparable between Paxlovid (19%) and placebo (21%), and most of them were mild in intensity [28].

Regarding the safety of oral drugs, we found that the total OR of adverse events in the drug and placebo groups was 0.85 (95% CI, 0.72–1.02), exhibiting no significant difference in the incidence of adverse events between the drug group and the placebo group. This indicates that the oral drugs did not improve the adverse events, and also they did not aggravate the occurrence of adverse events, i.e. the oral antiviral drugs are generally safe. The most common adverse events of the three oral antiviral drugs include nausea, diarrhoea, headache, runny nose and muscle pain. These studies show that most of the adverse events after taking oral antiviral drugs are mild, and few serious adverse events have been reported [18,28,29]. In contrast, existing vaccines have shown increasingly more severe adverse reactions. In addition to common diarrhoea, arthralgia and infra-red radiation at the injection site, rare serious adverse events including allergy, deep vein thrombosis and pulmonary embolism have been reported, although these adverse events are rare. However, adverse events have been reported in genetic vaccines BNT162b2 (Pfizer BioNTech) and gene-1273 (Moderna, Cambridge, MA), as well as adenovirus vector vaccines ChAdOx1 nCOV-19 (AstraZeneca, Cambridge, UK) and Ad26 [30,31]. Besides, studies have shown that even if vaccinated humans are infected with novel coronavirus variants, existing vaccines may not be effective against them [4,5], and reinfection of mutants with different antigenicities may reduce the overall efficacy of spike-based COVID-19 vaccines [30–33]. Oral antiviral drugs are probably safer than COVID-19 vaccine in general, although no clinical studies have compared their efficacies. Other advantages of oral drugs, such as molnupiravir and Paxlovid are that they can be produced in a large scale, do not require refrigerated shipping, do not require hospital administration, and are less expensive than other EUA approved COVID-19 vaccines and monoclonal antibodies [22,27].

Although our results are consistent with the clinical trials on COVID-19 drugs, there are still some shortcomings in this study. First, we included only English studies; many non-English studies were omitted. Second, no specific data was available for analysis, such as gender and age in the included literature, so no subgroup analysis was conducted in this respect. Moreover, the effect of confounding factors such as age and gender on the research results cannot be excluded. Besides, only one study was found on Paxlovid, which is insufficient for subgroup analysis. It can only be said that this clinical study shows that Paxlovid can effectively reduce the mortality or hospitalization rate of patients. Whether Paxlovid can effectively reduce mortality or hospitalization, the sample size of the study needs to be further expanded to obtain more scientific results. At present, the clinical study of this drug is still in progress, and it is expected to provide strong evidence in the future.

## Conclusions

This study shows that three novel oral antiviral drugs (molnupiravir, fluvoxamine and Paxlovid) are effective in reducing the mortality and hospitalization rates in COVID-19 patients. In addition, our study showed that the three oral antiviral drugs did not increase the occurrence of adverse events, thus exhibiting good overall safety. These three oral antiviral drugs are still being studied, and the available data suggest that they will bring new hope for COVID-19 recovery and have the potential to be a breakthrough and very promising treatment for COVID-19.

## Data Availability

The authors confirm that the data supporting the findings of this study are available within the article.

## References

[1] WHO. Coronavirus (COVID-19) data. [cited 2021 Nov 12]. Available from: https://www.who.int/data#reports

[2] Rivasi G, Bulgaresi M, Mossello E, et al. Course and lethality of SARS-CoV-2 epidemic in nursing homes after vaccination in Florence, Italy. Vaccines (Basel). 2021;9(10):1174. 34696282 10.3390/vaccines9101174 PMC8537408

[3] National health commission of the people's republic of China. [cited 2021 Nov 12]. Available from: http://www.nhc.gov.cn/jkj/s7915/202111/fc017abab2af4dfbbe5b4f0bdcbf8795.shtml 10.46234/ccdcw2021.270 PMC8855077 35186365

[4] Imran M, Kumar Arora M, Asdaq SMB, et al. Discovery, development, and patent trends on molnupiravir: a prospective oral treatment for COVID-19. Molecules. 2021;26(19):5795. 34641339 10.3390/molecules26195795 PMC8510125

[5] Christie A, Mbaeyi SA, Walensky RP. CDC interim recommendations for fully vaccinated people: an important first step. JAMA. 2021;325(15):1501–1502. 33688914 10.1001/jama.2021.4367

[6] Vitiello A, Troiano V, La Porta R. What will be the role of molnupiravir in the treatment of COVID-19 infection? Drugs Ther Perspect. 2021;37(12):572–579. 10.1007/s40267-021-00879-2 PMC8570236 34754175

[7] Ferner RE, Aronson JK. Remdesivir in covid-19. BMJ. 2020;369:m1610. 32321732 10.1136/bmj.m1610

[8] Vitiello A, Ferrara F, Porta R. Remdesivir and COVID-19 infection, therapeutic benefits or unnecessary risks? Ir J Med Sci. 2021;190(4):1637–1638. 10.1007/s11845-020-02482-2 PMC7801863 33433843

[9] Menéndez-Arias L. Decoding molnupiravir-induced mutagenesis in SARS-CoV-2. J Biol Chem. 2021;297(1):100867. 34118236 10.1016/j.jbc.2021.100867 PMC8188802

[10] Zhao J, Guo S, Yi D, et al. A cell-based assay to discover inhibitors of SARS-CoV-2 RNA dependent RNA polymerase. Antiviral Res. 2021;190:105078. 33894278 10.1016/j.antiviral.2021.105078 PMC8059291

[11] Agostini ML, Pruijssers AJ, Chappell JD, et al. Small-Molecule antiviral β-d- N 4-Hydroxycytidine inhibits a Proofreading-Intact coronavirus with a high genetic barrier to resistance. J Virol. 2019;93(24):e01348–19. 31578288 10.1128/JVI.01348-19 PMC6880162

[12] Rosenke K, Hansen F, Schwarz B, et al. Orally delivered MK-4482 inhibits SARS-CoV-2 replication in the Syrian hamster model. Nat Commun. 2021;12(1):2295. 33863887 10.1038/s41467-021-22580-8 PMC8052374

[13] Sheahan TP, Sims AC, Zhou S, et al. An orally bioavailable broad-spectrum antiviral inhibits SARS-CoV-2 in human airway epithelial cell cultures and multiple coronaviruses in mice. Sci Trans Med. 2020;12(541):eabb5883. 10.1126/scitranslmed.abb5883 PMC7164393 32253226

[14] Fischer W, Eron JJ, Holman W, et al. Molnupiravir, an oral antiviral treatment for COVID-19. medRxiv. 2021.

[15] Mahase E. Covid-19: Molnupiravir reduces risk of hospital admission or death by 50% in patients at risk, MSD reports. BMJ. 2021;375:n2422. 34607801 10.1136/bmj.n2422

[16] Calusic M, Marcec R, Luksa L, et al. Safety and efficacy of fluvoxamine in COVID-19 ICU patients: an open label, prospective cohort trial with matched controls. Br J Clin Pharmacol. 2021. 10.1111/bcp.15126 PMC8653355 34719789

[17] Seftel D, Boulware DR. Prospective cohort of fluvoxamine for early treatment of coronavirus disease 19. Open Forum Infect Dis. 2021;8(2):ofab050. 33623808 10.1093/ofid/ofab050 PMC7888564

[18] Lenze EJ, Mattar C, Zorumski CF, et al. Fluvoxamine *vs.* placebo and clinical deterioration in outpatients with symptomatic COVID-19: a randomized clinical trial. JAMA. 2020;324(22):2292–2300. 33180097 10.1001/jama.2020.22760 PMC7662481

[19] Reis G, Dos Santos Moreira-Silva EA, Silva DCM, et al. Effect of early treatment with fluvoxamine on risk of emergency care and hospitalisation among patients with COVID-19: the TOGETHER randomised, platform clinical trial. Lancet Glob Health. 2022;10(1):e42–e51. 34717820 10.1016/S2214-109X(21)00448-4 PMC8550952

[20] Pfizer’s novel COVID-19 oral antiviral treatment candidate reduced risk of hospitalization or death by 89% in interim analysis of phase 2/3 EPIC-HR study. 2021. Available from: https://www.pfizer.com/news/press-release/press-release-detail/pfizers-novel-covid-19-oral-antiviral-treatment-candidate

[21] Hetero announces interim clinical results from phase III clinical trials of molnupiravir conducted in India. 2021. Available from: https://c19mp.com/hetero.html

[22] Singh AK, Singh A, Singh R, et al. Molnupiravir in COVID-19: a systematic review of literature. Diabetes Metab Syndr. 2021;15(6):102329. 34742052 10.1016/j.dsx.2021.102329 PMC8556684

[23] Toots M, Yoon JJ, Hart M, et al. Quantitative efficacy paradigms of the influenza clinical drug candidate EIDD-2801 in the ferret model. Trans Res. 2020;218:16–28. 10.1016/j.trsl.2019.12.002 PMC7568909 31945316

[24] Kabinger F, Stiller C, Schmitzová J, et al. Mechanism of molnupiravir-induced SARS-CoV-2 mutagenesis. Nat Struct Mol Biol. 2021;28(9):740–746. 34381216 10.1038/s41594-021-00651-0 PMC8437801

[25] Zhou S, Hill CS, Sarkar S, et al. β-d-N4-hydroxycytidine inhibits SARS-CoV-2 through lethal mutagenesis but is also mutagenic to mammalian cells. J Infect Dis. 2021;224(3):415–419. 33961695 10.1093/infdis/jiab247 PMC8136050

[26] Sukhatme VP, Reiersen AM, Vayttaden SJ, et al. Fluvoxamine: a review of its mechanism of action and its role in COVID-19. Front Pharmacol. 2021;12:652688. 33959018 10.3389/fphar.2021.652688 PMC8094534

[27] 8 Lingering questions about the new Covid pills from Merck and Pfizer. [cited 2021 Nov 16]. Available from: https://www.statnews.com/2021/11/15/8-lingering-questions-about-the-new-covid-pills-from-merck-and-pfizer/

[28] Pfizer seeks emergency use authorization for novel COVID-19 oral antiviral candidate. Available from: https://www.pfizer.com/news/press-release/press-release-detail/pfizer-seeks-emergency-use-authorization-novel-covid-19

[29] Painter WP, Holman W, Bush JA, et al. Human safety, tolerability, and pharmacokinetics of molnupiravir, a novel Broad-Spectrum oral antiviral agent with activity against SARS-CoV-2. Antimicrob Agents Chemother. 2021;65(5):e02428–20. 10.1128/AAC.02428-20 PMC8092915 33649113

[30] Pormohammad A, Zarei M, Ghorbani S, et al. Efficacy and safety of COVID-19 vaccines: a systematic review and Meta-Analysis of randomized clinical trials. Vaccines (Basel). 2021;9(5):467. 34066475 10.3390/vaccines9050467 PMC8148145

[31] Sharif N, Alzahrani KJ, Ahmed SN, et al. Efficacy, immunogenicity and safety of COVID-19 vaccines: a systematic review and Meta-Analysis. Front Immunol. 2021;12:714170. 34707602 10.3389/fimmu.2021.714170 PMC8542872

[32] Wibmer CK, Ayres F, Hermanus T, et al. SARS-CoV-2 501Y.V2 escapes neutralization by South African COVID-19 donor plasma. Nat Med. 2021;27(4):622–625. 33654292 10.1038/s41591-021-01285-x

[33] Garcia-Beltran WF, Lam EC, St Denis K, et al. Multiple SARS-CoV-2 variants escape neutralization by vaccine-induced humoral immunity. Cell. 2021;184(9):2372–2383.e9. 33743213 10.1016/j.cell.2021.03.013 PMC7953441

